# OAT在非小细胞肺癌中的表达及生物信息学分析

**DOI:** 10.3779/j.issn.1009-3419.2012.09.04

**Published:** 2012-09-20

**Authors:** 丹菲 周, 西安 程, 拴盈 杨, 宗娟 明, 维 李, 秋红 张, 玉萍 张

**Affiliations:** 1 710004 西安，西安交通大学附属第二医院呼吸内科 Derpartment of Respiratory Medicine, the Second Affiliated Hospital of Xi'an Jiaotong University, Xi'an 710004, China; 2 727000 铜川，铜川市人民医院 People's Hospital of Tongchuan City, Tongchuan 727000, China

**Keywords:** 鸟氨酸氨基转移酶, 肺肿瘤, 表达, 生物信息学, Ornithine aminotransferase (OAT), Lung neoplasms, Expression, Bioinformatics

## Abstract

**背景与目的:**

已有的研究表明，鸟氨酸氨基转移酶（ornithine aminotransferase, OAT）可能参与多种恶性肿瘤的发生和发展，本研究旨在检测非小细胞肺癌（non-small cell lung cancer, NSCLC）中OAT mRNA和蛋白质的表达，并对其进行生物信息学分析。

**方法:**

通过RT-PCR的方法比较A549和16HBE细胞间OAT mRNA水平的差异；采用免疫组织化学SP法检测55例肺癌组织和17例癌旁肺组织中OAT蛋白表达；利用生物信息学的方法对OAT蛋白的生物信息学特征和相互作用蛋白进行分析，并对筛选出来的相互作用蛋白进行GO注释和信号通路分析。

**结果:**

① A549中OAT mRNA相对含量较16HBE中低，两者差异约2.85倍。②NSCLC中OAT蛋白的表达明显高于癌旁肺组织（*P* < 0.05）；OAT蛋白在鳞癌和腺癌间的表达差异有统计学意义，而与患者性别、年龄、有无淋巴结转移、肿瘤直径及TNM分期无关。③生物信息学分析提示OAT蛋白定位于线粒体、为高度保守的亲水性蛋白，具有Aminotran-3结构域，可能存在多个丝/苏氨酸磷酸化位点，在信号转导、转录和分子转运等方面发挥一定的作用；在筛选出来的54种可能和OAT存在相互作用的蛋白质中，TNF和TRAF6这两种蛋白参与了NF-κB信号通路。

**结论:**

OAT可能在NSCLC的发生和发展中发挥重要作用，有望成为肺癌的早期诊断标志物和治疗的新靶点。

## Abstract (en)

**Background and objective:**

It has been proven that ornithine aminotransferase (OAT) might play an important role in the oncogenesis and progression of numerous malignant tumors. The aim of this study is to detect the mRNA and protein expression of OAT in non-small cell lung cancer (NSCLC), as well as to analyze the bioinformatic features and binary interactions.

**Methods:**

OAT mRNA expression was detected in A549 and 16HBE cell lines by reverse transcription-polymerase chain reaction. OAT protein expression was determined in 55 cases of NSCLC and 17 cases of adjacent non-tumor lung tissues by immunohistochemical staining. The bioinformatic features and binary interactions of OAT were analyzed. Gene ontology annotation and signal pathway analysis were performed.

**Results:**

OAT mRNA expression in A549 cells was 2.85-fold lower than that in 16HBE cells. OAT protein expression was significantly higher in NSCLC tissues than that in adjacent non-tumor lung tissues. A significant difference of OAT protein expression was existed between squamous cell lung cancer and adenocarcinoma (*P* < 0.05), but was not correlated with the gender, age, lymph node metastasis, tumor size, and TNM stages. Bioinformatic analysis suggested that OAT was a highly homologous and stable protein located in the mitochondria. An aminotran-3 domain and several sites of phosphorylation, which may function in signal transduction, gene transcription, and molecular transit, were found. In the 54 selected binary interactions of OAT, TNF and TRAF6 play roles in the NF-κB pathway.

**Conclusion:**

OAT may play an important role in the oncogenesis and progression of NSCLC. Thus, OAT may be a novel biomarker for the diagnosis of NSCLC or a new target for its treatment.

肺癌是目前世界上发病率和死亡率最高的恶性肿瘤^[1]^。因其缺乏有效的早期诊断方法^[2]^，70%-80%的患者确诊时已是肿瘤中晚期，存在淋巴结转移或远处转移。虽然目前的治疗方式不断进步和发展，但是肺癌患者的预后仍不尽人意，5年和10年生存率分别为14%和8%^[3, 4]^。早期诊断和早期治疗是改善患者预后、降低肺癌死亡率的关键。应用蛋白质组学的方法筛选肺癌早期诊断标志物和治疗靶点是近期研究的热点，但是至今尚无理想的生物标志物。本课题前期通过线粒体蛋白质组学研究，筛选出一系列差异蛋白。其中鸟氨酸氨基转移酶（ornithine aminotransferase, OAT）在肺腺癌A549细胞和正常支气管上皮细胞16HBE间的表达差异超过2倍。通过文献复习，进一步发现OAT可能和肝细胞癌、胃癌、前列腺癌等其它肿瘤也存在相关性，而其与非小细胞肺癌（non-small cell lung cancer, NSCLC）的相关研究尚未见报道。本研究将进一步利用细胞和组织标本分别对OAT mRNA和OAT蛋白的表达进行检测，以验证前期线粒体蛋白质组学的结果并分析其临床意义。此外，利用生物信息学方法对OAT蛋白进行初步分析和相互作用蛋白预测，为进一步探究其在NSCLC发生和发展中的作用机制奠定基础。

## 1 材料与方法

### 1.1 细胞及组织来源

人肺腺癌细胞株A549来自西安交通大学中心实验室保存；人正常支气管上皮细胞株16HBE购于中国医学科学院肿瘤细胞库；55例肺癌组织标本取自西安交通大学医学院第二附属医院2006年-2011年手术病例，其中17例同时收集癌旁 > 5 cm处肺组织标本，其中男性35例，女性20例，鳞癌35例，腺癌20例，27例无淋巴结转移，28例存在淋巴结转移，平均年龄为（58.3±10）岁。根据国际抗癌联盟2009年第7版TNM分期标准：Ⅰ期20例，Ⅱ期15例，Ⅲ期18例，Ⅳ期2例。所有入选病例术前未经放、化疗，无肝硬化、自身免疫病等合并症，术后经病理确诊为肺癌。组织标本经福尔马林固定，常规石蜡包埋、切片备用。

### 1.2 主要试剂

RPMI1640培养液、胎牛血清、青-链霉素溶液（美国Hyclone）；0.25%胰蛋白酶溶液（上海碧云天）；RNAfast200总RNA极速抽提试剂盒（上海飞捷）；RevertAid^TM^ First Strand cDNA Synthesis Kit（美国Fermentas）；OAT及actin-beta引物（上海生工）；PCR mix（北京康为世纪）；溴化乙锭溶液、DNA Marker I、焦碳酸二乙酯（北京天根生化）；琼脂糖粉（西班牙Biowest）；兔抗人OAT多克隆抗体（美国Abcam）；免疫组化染色试剂盒、浓缩型DAB试剂盒、中性树胶（北京中杉金桥）；其它实验试剂均为国产分析纯。

### 1.3 细胞培养及RT-PCR

A549和16HBE细胞用含10%FBS的RPMI 1640培养液，于37 ℃、5%CO_2_培养箱中培养2 d-3 d后用0.25%胰酶消化传代。取对数生长期细胞，按RNAfast200试剂盒步骤进行细胞总RNA的提取。利用RNA电泳检测其完整性，紫外分光光度法测定OD_260_/OD_280_值进行RNA定量。cDNA合成按RevertAid^TM^ First Strand cDNA Synthesis Kit操作说明进行：在PCR管中加入2 μL总RNA，1 μL随机引物，再加入RNase free H_2_O至总体积为12 μL，混合均匀后经65 ℃加热5 min，迅速置于冰上，再依次加入5×Reaction buffer 4 μL、RNase inhibitor 1 μL、10 mM dNTP mix 2 μL、RevertAid^TM^ M-MuLV Reverse Transcriptase 1 μL使总体积为20 μL，震荡混匀离心后行逆转录反应，条件为：42 ℃ 60 min，70 ℃ 5 min，最后终止反应得到cDNA产物，-20 ℃保存备用。PCR过程：将目的基因的退火温度从48 ℃-60 ℃之间每隔1 ℃设立温度梯度进行扩增，并对模板cDNA浓度、引物量及循环数等进行适当调整，最终确立最佳PCR反应条件。

OAT的PCR扩增体系如下：1:100稀释后cDNA 1 μL，上下游引物各1 μL，2×PCR Mix 12.5 μL，ddH_2_O 9.5 μL，总体积25 μL。PCR反应条件：94 ℃、3 min；94 ℃、30 s，52 ℃、30 s，74 ℃、45 s，循环35次；74 ℃、5 min。

Actin-beta的PCR扩增体系如下：1:1, 000稀释后cDNA 1 μL，上下游引物各1 μL，2×PCR Mix 12.5 μL，ddH_2_O 9.5 μL，总体积25 μL。PCR反应条件：94 ℃、3 min；94 ℃、30 s，52 ℃、30 s，74 ℃、45 s，循环35次；74 ℃、5 min。

PCR产物进行常规电泳并采集凝胶图像，利用Gel-Pro专业图像分析软件，测定各条带的积分光密度值（integrated optical density, IOD），以actin-beta为内参，计算A549和16HBE细胞系中OAT mRNA的相对含量。上述实验重复3次。

### 1.4 组织化学染色

过程如下：脱蜡和水化、微波枸橼酸抗原修复、3% H_2_O_2_孵育、正常山羊血清封闭、一抗（OAT 1:30稀释）4 ℃过夜、二抗孵育、辣根酶标记链霉卵白素孵育、DAB显色、苏木素复染、脱水、透明并封片。用已知阳性片作为阳性对照，PBS代替一抗作为阴性对照。

免疫组织化学结果判定^[3]^：由两位病理医师采取双盲法进行判断，OAT蛋白以胞浆中出现棕黄色颗粒为阳性，每例随机观察5个高倍镜视野。采用半定量积分法判定结果，根据阳性细胞所占百分比（< 5%：0分；5%-24%：1分；25%-50%：2分； > 50%：3分）和染色强度（未着色：0分；淡黄色：1分；中度黄色：2分；棕黄色：3分）进行评分。将两种分值相加：0分为“-”，1分-2分为“+”，3分-4分为“++”，5分-6分为“+++”。我们将“-”定义为阴性，将“+、++、+++”定义为阳性表达。

### 1.5 统计学方法

所有数据应用SPSS 16.0软件进行处理，采用χ^2^检验分析组间差异。*P* < 0.05为差异有统计学意义。

### 1.6 生物信息学分析

利用NCBI数据库对OAT mRNA、蛋白质序列进行搜索，在此基础上，利用ProtParam、BLAST、SOPMA、TMpred、SMART、WoLF PSORT、SWISS-MODEL Repository、NetPhos 2.0 Server^[4]^和ProtFun 2.2 Server^[5]^等在线工具和软件对蛋白质的理化性质、蛋白同源性、二级结构、跨膜结构域、功能结构域、亚细胞定位、三维结构、磷酸化位点和功能等进行初步分析。利用IntAct、APID、MINT、DIP、STRING等蛋白质相互作用在线数据库，分析预测可能和OAT存在相互作用的蛋白。在DAVID（The Database for Annotation, Visualization, and Integrated Discovery）平台上对筛选出来的相互作用蛋白进行GO（Gene Ontology）功能注释和信号通路分析。

## 2 结果

### 2.1 细胞总RNA定量及完整性鉴定

由A549所提取细胞总RNA的OD_260_/OD_280_值在1.95-2.07之间，RNA浓度平均值为255.9 ng/μL。16HBE细胞总RNA的OD_260_/OD_280_值在1.92-2.04之间，RNA浓度平均值为374.1 ng/μL，表明所提取总RNA的纯度较高。1%琼脂糖凝胶电泳清晰可见28S、18S两条带（图 1），无明显杂带或拖尾现象，且28S亮度约为18S的2倍，表明所提取总RNA的完整性较好，基本无降解。

**1:**
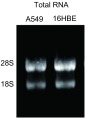
细胞提取总RNA电泳结果 Electrophoresis of total RNA extracted from cell lines

### 2.2 RT-PCR结果

*OAT*基因在A549细胞和16HBE细胞中的PCR产物电泳结果如图 2所示，肉眼可见A549组所对应条带与16HBE组相比较暗，经Gel-Pro软件测定各条带的积分光密度值（IOD），以actin-beta为内参，计算A549和16HBE细胞株中*OAT*基因mRNA的相对含量分别为0.20±0.05和0.57±0.07，A549组中*OAT*基因mRNA的相对含量较低，两者差异约为2.85倍。

**2:**
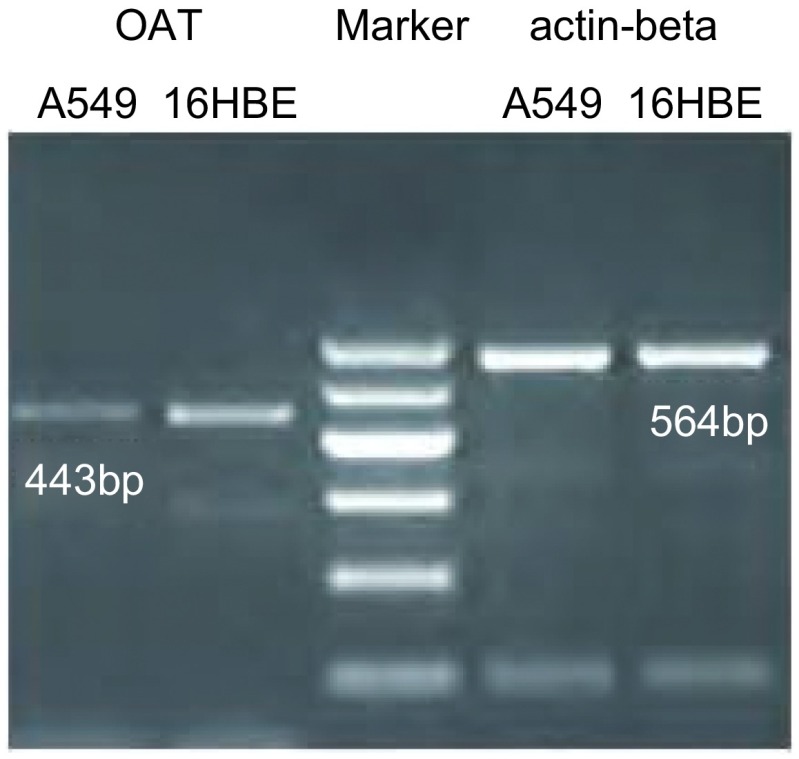
OAT基因PCR电泳结果 Electrophoresis of OAT gene PCR product

### 2.3 免疫组化结果

OAT蛋白以棕黄色颗粒表达于胞浆，在NSCLC组织中表达阳性率为81.82%（45/55），而在癌旁肺组织中均无阳性表达（χ^2^=39.080, *P* < 0.001），差异有统计学意义（图 3）。OAT表达为“-”、“+”、“++”和“+++”的分别有10例、27例、10例和8例（图 4）。按性别、年龄、组织病理学类型、有无淋巴结转移、肿瘤直径及TNM分期对NSCLC进行分组分析，结果显示OAT蛋白在腺癌和鳞癌组间的表达差异有统计学意义（χ^2^=5.169, *P*=0.023）。OAT蛋白在肺腺癌组中的阳性率（100%）高于其在肺鳞癌组中的阳性率（71.43%）。而OAT蛋白的表达和患者性别、年龄、有无淋巴结转移、肿瘤直径及TNM分期无关。

**3:**
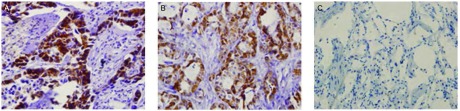
OAT蛋白在肺鳞癌（A）、肺腺癌（B）和癌旁肺组织（C）中的表达（IHC，×400） The expression of OAT in squamous lung cancer (A), adenocarcinoma (B) and adjacent non-tumor lung tissue (C) (IHC, ×400)

**4:**
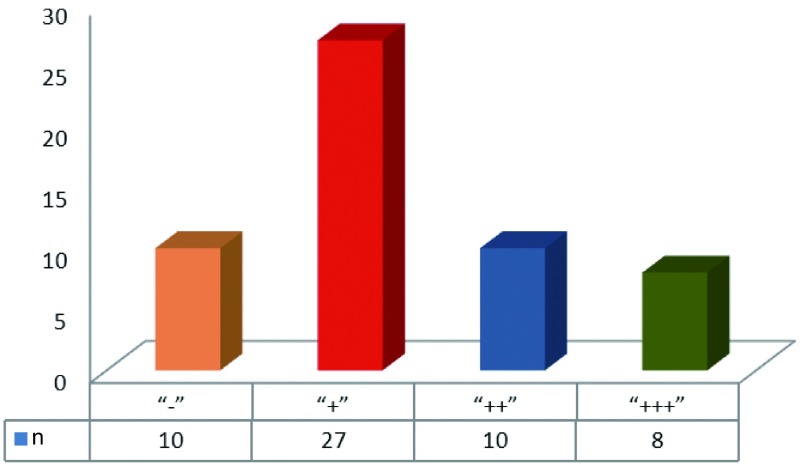
OAT蛋白在肺癌组织中的表达情况示意图 The expression of OAT in lung cancer tissues

### 2.4 生物信息学结果

*OAT*基因具有两个mRNA转录本，分别编码两个不同的线粒体亚型蛋白；该基因在人类、猩猩、黑长臂猿、小鼠、大鼠、大熊猫和牛等物种间高度保守，具有较高的同源性；OAT蛋白分子量为48, 534.8，理论pI为6.57，主要由亮氨酸（10.5%）、丙氨酸（8.4%）、甘氨酸（8.2%）和缬氨酸（8.0%）等组成，是一种偏向亲水性的，较为稳定的蛋白质；其二级结构主要包含α-螺旋（42.14%）和无规则卷曲（29.16%）（图 5）；TMpred结构域分析软件显示OAT蛋白可能并不存在跨膜结构域；SMART数据库搜索结果提示该蛋白具有结合磷酸吡哆醛和氨基转移活性的aminotran-3结构域；OAT蛋白的亚细胞定位预测也证实了本研究前期实验中线粒体蛋白的指向；NetPhos 2.0 Server预测磷酸化位点结果提示OAT蛋白可能存在7个丝氨酸磷酸化位点，7个苏氨酸磷酸化位点和9个酪氨酸磷酸化位点（图 6）；图 7所示是OAT的三维结构图，它是利用SWISS-MODEL Repository的搜索功能，并整合SMTL、RCSB、PDBe、SCOP和CATH数据库中的相关数据所得。初步对OAT蛋白的功能预测提示其可能在信号转导、转录和转运等方面具有一定的作用（表 1）。

**5:**
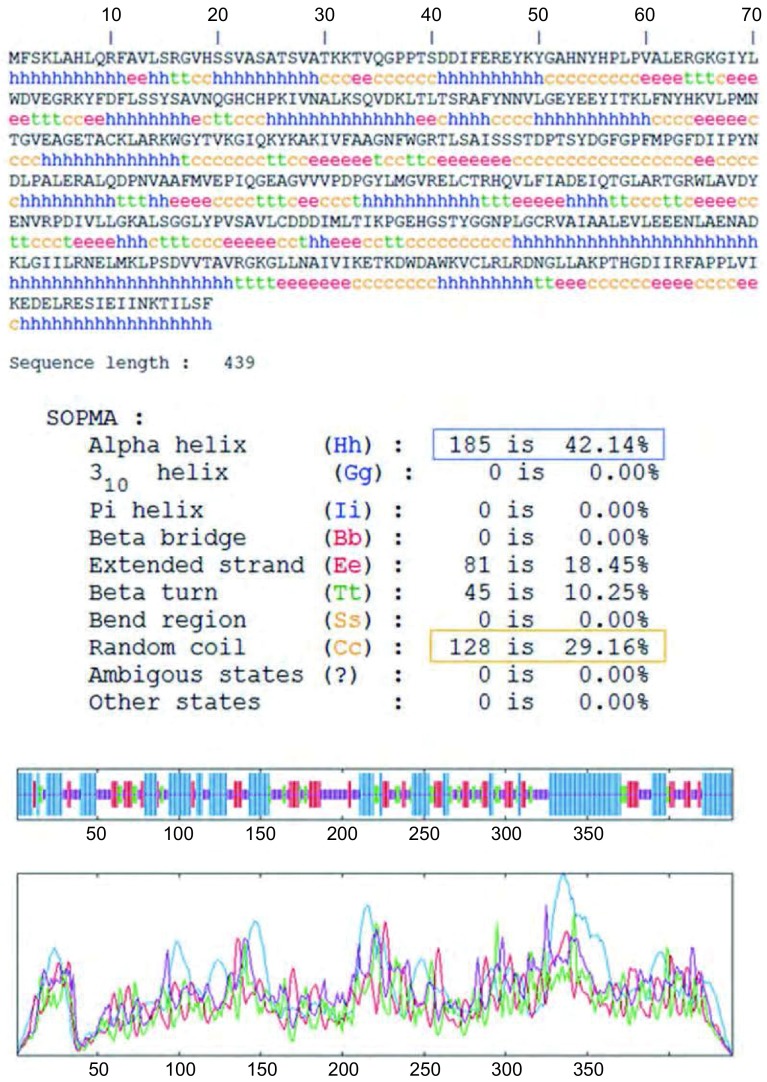
SOPMA软件对OAT二级结构预测图 The secondary structure prediction result of OAT by SOPMA

**6:**
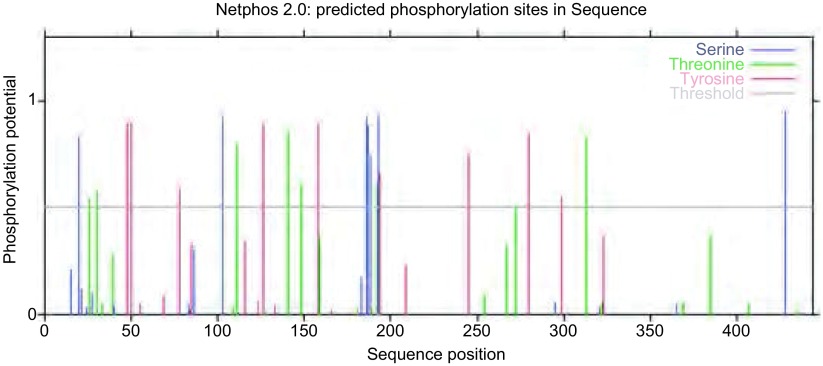
OAT蛋白质的磷酸化位点预测结果 The result of phosphorylation sites prediction of OAT

**7:**
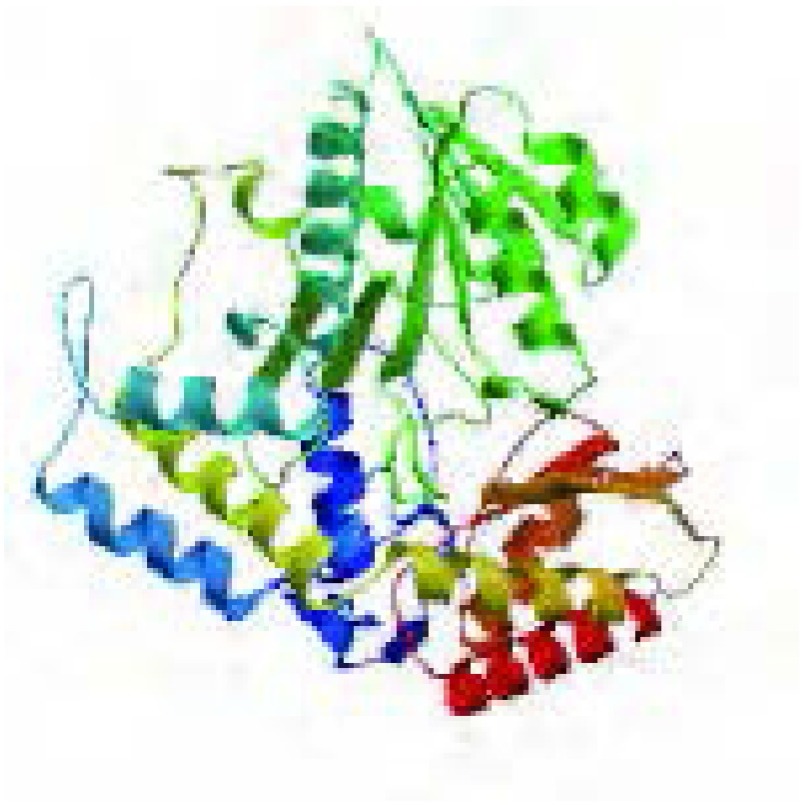
OAT蛋白质三维结构图 The three-demensional structure of OAT protein

**1:** *OAT*基因功能初步分析结果 The functional prediction of *OAT* Gene

OAT Gene Ontology category	Prob	Odds
Signal_transducer	0.096	0.447
Receptor	0.007	0.041
Hormone	0.001	0.206
Structural_protein	0.002	0.073
Transporter	0.025	0.227
Ion_channel	0.010	0.168
Voltage-gated_ion_channel	0.004	0.170
Cation_channel	0.010	0.215
Transcription	0.027	0.215
Transcription_regulation	0.018	0.144
Stress_response	0.013	0.144
Immune_response	0.013	0.151
Growth_factor	0.006	0.421
Metal_ion_transport	0.009	0.020

通过搜索IntAct、MINT、DIP、InteroPorc和STRING等在线数据库，发现存在重复和冗余数据，为了获得相对可靠的相互作用蛋白，进一步将具有文献依据的相互作用蛋白总结如下（表 2），表中蛋白质相互作用关系的获取方式有：免疫共沉淀、串联亲和纯化和酵母双杂交等。

**2:** 具有文献依据的OAT相互作用蛋白及相关信息 The binary interactions of OAT in literature

Molecular A	Molecular B	First author (year)	Method
OAT	HLA-B、PTP4A3、BB1、EIF1B、ARF6、SSSCA1、PRKAB1、MCC、CCNA1、EIF6、TRAF6	Ewing *et al*. (2007)	Anti bait coimmunoprecipitation
OAT	MYC	Koch *et al*. (2007)	Tandem affinity purification
OAT	MAP1LC3A	Behrends *et al*. (2010)	Anti tag coimmunoprecipitation
OAT	C2orf18	Stelzl *et al*. (2005)	Two hybrid pooling approach
OAT	DMWD、OTUD4	Sowa *et al*. (2009)	Anti tag coimmunoprecipitation
OAT	UBQLN4	Lim *et al*. (2006)	Two hybrid
OAT	CLK4、GLUT-4、CHEK1、NMNAT1、DDX49、PIN1、CSNK1E、SIK1、CSNK1G3、HSPA6、HSP90AB1、NMNAT3	Michaut *et al*. (2008)	Interologs mapping
OAT	MPPB	Kitada S（2007）	
OAT	OTC、SLC25A2、SLC25A15	Vastrik *et al*. (2007)	
OAT	DIPP	Vinayagam A (2011)	Two hybrid
OAT	ICT1/DS1	Richter R (2010)	Anti tag coimmunoprecipitation

STRING数据库的结果（图 8，图中着色节点表示和OAT可能直接相关的分子，白色节点代表可能存在更深层次关系的分子，节点间连线以不同的颜色分别代表不同的依据来源，下方表中详细罗列了各着色节点的相关信息及综合评分）则进一步扩大了相互作用的预测范围，该数据库包含了直接（物理上）和间接（功能上）的各种相互作用关系，数据结果综合了基因背景、高通量实验、共表达和已知信息等，并对每一个相互作用关系进行评分，该数据库目前涵盖了来自1133个物种的5, 214, 234种蛋白质的相互作用信息。STRING数据库预测所得与OAT相互作用蛋白中评分最高的20种蛋白依次为：OTC、ARG1、ARG2、ALDH18A1、ODC1、ALDH4A1、ASS1、ASL、ACY1、TBC1D25、TNF、TLR4、SLC22A6、SLC22A8、IL2、SLC22A7、B3GAT1、GLUD2、GLUL、SCL22A20。

**8:**
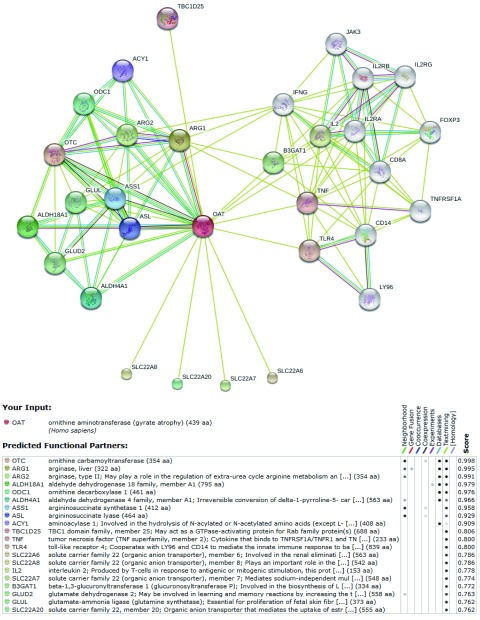
STRING数据库：OAT相互作用蛋白预测结果 Predicted functional partners of OAT in STRING database

综合各数据库信息，筛选出54种可能和OAT存在相互作用的蛋白质并提交至DAVID在线平台，其中52种蛋白质在DAVID中查找到对应的ID，通过Gene Ontology对这52种蛋白质进行本体论注释，在相应参数设置条件下，发现共有35种蛋白质参与了73种不同的生物学途径（biological process），20种蛋白质参与了14种不同的细胞组件（cellular component），27种蛋白质参与了18种不同的分子功能（molecular function）。分子信号通路分析结果（图 9）显示有17种蛋白质参与了包括：NF-κB信号通路、NOD样受体信号通路及精氨酸和脯氨酸代谢等在内的8个重要的信号转导通路。

**9:**
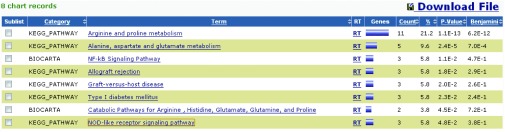
17种蛋白质基于KEGG和BIOCARTA数据库的信号转导通路分析结果 The signal pathway analysis of 17 proteins based on KEGG and BIOCARTA databases

## 3 讨论

OAT蛋白由位于染色体10q26的*OAT*基因编码，以同源四聚体的形式存在于线粒体基质，存在两种同工酶，分别为肝型和肾型。在OAT的催化作用下，鸟氨酸可转化为谷氨酸，后者可进一步生成脯氨酸和谷氨酰胺，在氨基酸的生物合成和转化过程中发挥着重要作用。该酶首先以前体的形式在细胞质中合成，转运进入线粒体后裂解为成熟的OAT蛋白质。*OAT*基因遗传缺陷可导致常染色体隐性遗传疾病——脉络膜视网膜环状萎缩^[6]^。对于OAT和肿瘤的相关研究，前期多为检测动物模型或细胞中该酶活性的改变。其中关于肝细胞肿瘤^[7, 8]^、下颌下腺癌^[7]^、淋巴瘤^[8]^等的相关研究提示：随着肿瘤的发生和发展，OAT的酶活性明显下降。但是近年来有研究显示在Morris肝细胞瘤中OAT的含量较正常肝脏高15倍^[9]^、肝癌组织中的*OAT*基因定量高于非肝癌组织^[10]^，且可能是肝脏β-catenin信号通路^[11]^及前列腺癌中雄激素受体^[12]^的靶向基因。此外，研究还发现OAT是肿瘤细胞纺锤体组装的必需成分，通过抑制OAT，可以干扰细胞有丝分裂，诱导肿瘤细胞死亡^[13]^。近期，Miyasaka等^[14]^在采用238pu α粒子照射人支气管上皮细胞BEP2D进行体外转化的过程中发现，在转化早期R15H20细胞中发现OAT蛋白高表达。Cadoret等^[15]^在诱导胰腺癌细胞中CapG蛋白高表达的同时也检测到了OAT蛋白的表达上调。Jariwala等^[16]^在转移性犬乳腺癌中发现OAT蛋白表达上调超过1.5倍。Wang等^[17]^通过蛋白质组学和RT-PCR的研究结果均表明：经过己烯雌酚处理后的前列腺癌细胞中OAT表达上调，此外，还有多个凋亡和氧化应激相关的线粒体蛋白质在经历己烯雌酚处理后也发生了表达量的变化。由此，研究者推测己烯雌酚诱导的氧化应激可能导致细胞凋亡。但对于OAT和其它相关蛋白的确切功能尚不清楚，有待进一步研究。

本实验通过体外细胞培养，在转录水平对*OAT*基因在不同细胞间的表达差异进行比较，结果显示肺腺癌细胞中*OAT*基因mRNA相对含量低。而免疫组化进行蛋白表达差异的比较，发现NSCLC中OAT蛋白表达显著高于癌旁肺组织，这个结果和前期线粒体蛋白质组学的结果一致。对于*OAT*基因在转录和翻译水平不一致的原因分析可能为：①在mRNA到蛋白质表达的过程中，存在转录后、翻译及翻译后各环节的精细调控，涉及mRNA的出核过程、细胞浆定位、稳定性，蛋白质翻译和翻译后水解、加工等，这些都可能造成转录物和蛋白质的丰度不一致^[18, 19]^；②体外细胞培养和患者手术组织标本之间的差异所致；③RT-PCR和免疫组化在定量上都无法达到精确的程度，这是实验方法本身存在的缺陷；④实验重复次数及标本量少所造成的局限。

本研究中*OAT*基因在NSCLC和对照组间的表达差异是肯定的，具有统计学意义，而肺腺癌组织中OAT蛋白的表达高于肺鳞癌。虽然肺腺癌和鳞癌都起源于支气管上皮细胞，但两者在组织形态和生物学行为方面仍存在较大的差异。肿瘤的形成是在多种因素的影响下发生的细胞癌变，本实验结果显示OAT在肺腺癌中的表达高于肺鳞癌，提示OAT的表达可能与细胞病理类型的分化相关，对NSCLC的病理分型、治疗方案选择和预后判断可能有一定的价值。此外，OAT蛋白表达与患者TNM分期、有无淋巴结转移、肿瘤直径等临床病理特征无关，提示其可能是NSCLC形成过程中的早期事件。由此，我们认为OAT有望成为NSCLC的早期诊断标志物和治疗的新靶点。

为进一步研究OAT在NSCLC中的功能和作用机制，我们期望通过相互作用蛋白的筛选来对OAT的功能进行初步阐述。目前研究蛋白质相互作用的实验技术方法主要有酵母双杂交、哺乳动物细胞双杂交、免疫共沉淀、荧光共振能量转移、串联亲和纯化、蛋白质芯片和质谱等，这些技术在蛋白质相互作用领域做出了重大的贡献，但是实验研究也存在一定的缺陷，通常需要特殊的实验设备和条件，操作过程复杂，需要大量资金和人力的投入。生物信息学是在生命科学的研究中，综合了计算机科学和应用数学的方法而形成的一门新兴学科。在基因组和蛋白质组研究获得了大量实验数据的基础上，生物信息学在数据的储存、分析、检索和共享等层面发挥了不可替代的作用。本研究通过整合多种数据库和软件功能，在对OAT进行初步分析的基础上预测其相互作用蛋白并分析可能参与的生物过程和信号转导通路。其中，TNF和TRAF6是两种可能和OAT存在相互作用的蛋白，它们参与了NF-κB信号转导通路，该通路是目前已知的重要的肿瘤相关信号转导通路。此外，其他可能和OAT存在相互作用蛋白多涉及物质合成、肿瘤信号转导及生长调控等重要生物过程。这间接提示了OAT蛋白可能通过参与多种信号转导通路，从而在NSCLC的发生和发展中发挥重要的作用，为深入研究其在NSCLC中的作用机制提供了一个新的思路。
